# Sex differences in the combined influence of inflammation and nutrition status on depressive symptoms: insights from NHANES

**DOI:** 10.3389/fnut.2024.1406656

**Published:** 2024-05-29

**Authors:** Yifeng Xu, Zhaoqi Yan, Liangji Liu

**Affiliations:** ^1^School of Clinical Medicine, Jiangxi University of Chinese Medicine, Nanchang, Jiangxi, China; ^2^Affiliated Hospital of Jiangxi University of Chinese Medicine, Nanchang, Jiangxi, China

**Keywords:** sex differences, advanced lung cancer inflammation index, depression, National Health and Nutrition Examination Survey, a cross-sectional study

## Abstract

**Background:**

Both nutrition and inflammation are associated with depression, but previous studies have focused on individual factors. Here, we assessed the association between composite indices of nutrition and inflammation and depression.

**Methods:**

Adult participants selected from the National Health and Nutrition Examination Survey (NHANES) between 2005 and 2018 were chosen. The exposure variable was the Advanced Lung Cancer Inflammation Index (ALI) integrating nutrition and inflammation, categorized into low, medium, and high groups. The outcome variable was depression assessed using the Patient Health Questionnaire-9 (PHQ-9). A multivariable logistic regression model was employed to evaluate the relationship between ALI and the risk of depression.

**Results:**

After extensive adjustment for covariates, in the overall population, participants with moderate and high levels of ALI had a decreased prevalence of depression compared to those with low ALI levels, with reductions of 17% (OR, 0.83; 95% CI: 0.72–0.97) and 23% (OR, 0.77; 95% CI: 0.66–0.91), respectively. Among females, participants with moderate and high ALI levels had a decreased prevalence of depression by 27% (OR, 0.73; 95% CI: 0.60–0.88) and 21% (OR, 0.79; 95% CI: 0.64–0.98), respectively, compared to those with low ALI levels, whereas no significant association was observed among males. Subgroup analyses based on females and males yielded consistent results.

**Conclusion:**

In this study, we observed a negative correlation between moderate to high levels of ALI and the prevalence of depression, along with gender differences. Specifically, in females, greater attention should be given to the nutritional and inflammatory status.

## Introduction

Depression is a common and serious mental disorder (1), affecting over 300 million people worldwide (2). Its features include feelings of sadness, emptiness, cognitive impairment, and decreased energy (3), ranking among the top three global causes of disability (4). Moreover, its prevalence has significantly increased due to the previous COVID-19 pandemic (5), leading to substantial socio-economic burdens. Furthermore, depression is a recurrent chronic disease associated with increased mortality rates in cardiovascular, metabolic, and cancer diseases (6). Therefore, finding effective prevention methods or assessment indicators is crucial.

So far, the etiology and mechanisms of depression still lack a precise definition to date (7). It has been reported that inflammation, oxidative stress, hypothalamic–pituitary–adrenal (HPA) axis dysregulation, and depression may be physiopathologically linked (8). Elevated inflammatory signaling disrupts neurotransmitter metabolism, alters neural activity in mood-related brain regions, and impairs neuronal health (9, 10). Additionally, the release of pro-inflammatory cytokines leads to HPA axis dysregulation, triggering neurotoxin synthesis, resulting in oxidative stress and enhanced neurodegeneration (11). Furthermore, a meta-analysis supports the beneficial effects of anti-inflammatory treatment on depression (12). Malnutrition is also one of the risk factors for exacerbating depression and depressive symptoms (13, 14); studies have shown that compared to individuals with adequate nutrition, those with malnutrition have a 3-fold increased risk of developing depression (15), while a meta-analysis of 13 epidemiological studies indicated that healthy dietary patterns were associated with a 16% reduction in the risk of depression (16). Of course, some nutrients such as tryptophan and vitamin B6 also play crucial roles in the neuroendocrine system (17). Therefore, a comprehensive assessment of the combined impact of inflammation and nutrition on patients with depression is necessary.

Currently, research on inflammation and nutritional assessment in depression mostly focuses on one factor without simultaneously considering inflammation and nutritional status. The Advanced Lung Cancer Inflammation Index (ALI), calculated by multiplying body mass index (BMI) by serum albumin and then dividing by the neutrophil-to-lymphocyte ratio (NLR), comprehensively evaluates inflammation and nutritional status. In recent years, it has been not only applied in lung cancer patients but also shown close associations with the prognosis of hypertension and heart failure (18, 19), making it clinically practical due to its calculation from objective parameters obtained through laboratory tests. Additionally, women are at a higher risk of depression, yet no studies have explored gender differences in the risk of depression considering inflammation and nutrition. This study, based on the National Health and Nutrition Examination Survey (NHANES) database, aims to assess the association between inflammation and nutritional status and the risk of depression, further exploring gender differences therein.

## Materials and methods

### Data source and participants

The NHANES survey, led by the Centers for Disease Control and Prevention (CDC), employs a complex multi-stage probability sampling design and is a nationally representative survey aimed at assessing the health and nutritional status of adults and children in the United States. Currently, data collection and dissemination occur every 2 years as a cycle by the CDC’s National Center for Health Statistics (NCHS), encompassing demographic, dietary, examination, laboratory, and questionnaire data. The NHANES research protocol has been approved by the Research Ethics Review Board of the National Center for Health Statistics, and written informed consent has been obtained from all participants.

**Figure 1:**
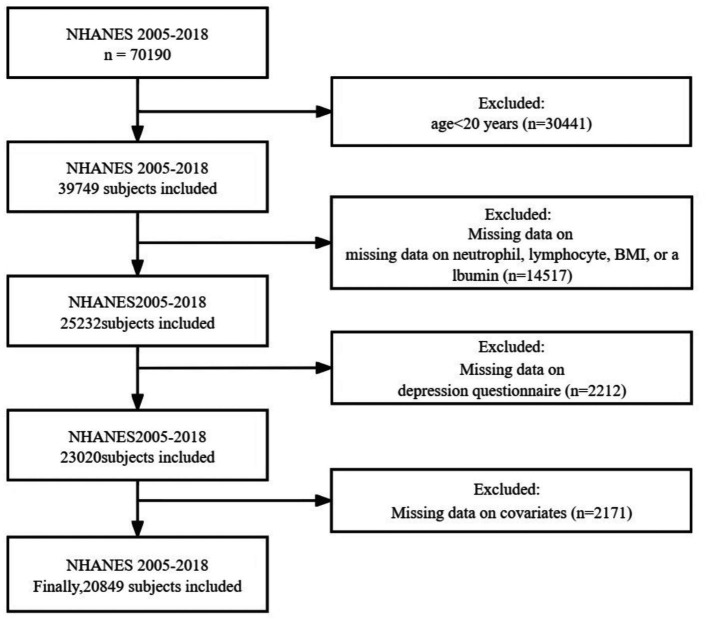
Flow chart of this study.

For this cross-sectional study, we extracted relevant data from a total of seven NHANES cycles spanning from 2005 to 2018 as the basis for analysis. Furthermore, since the collection of adult education levels in NHANES is completed among participants aged 20 and older, we have selected adults aged 20 and above for our study. Participants were excluded based on the following criteria (Figure 1): (1) individuals under the age of 20; (2) individuals with missing data on neutrophil, lymphocyte, BMI, or albumin, which are required for calculating ALI; (3) individuals with missing data on depression questionnaire; and (4) individuals with missing covariate data. Ultimately, a total of 20,849 individuals met the inclusion criteria for this study.

### Assessment of ALI

The comprehensive impact of inflammation levels and nutritional status on depression was assessed using the ALI. The calculation formula for ALI is as follows: ALI = BMI (kg/m^2^) × albumin level (g/dL)/NLR, where NLR is derived from the ratio of neutrophil count to absolute lymphocyte count. Due to the right-skewed distribution of ALI, log2 transformation was applied during the regression analysis (Supplementary Figure S1). Based on log2(ALI) levels, participants were divided into three groups: low (≤5.6527), medium (>5.6527 and ≤ 6.1931), and high (>6.1931) tertiles.

### Assessment of depressive symptoms

Depressive symptoms were assessed using the Patient Health Questionnaire (PHQ-9), a screening tool consisting of nine items, commonly used to assess the frequency of depressive symptoms over the past 2 weeks. The PHQ-9 total score ranges from 0 to 27, with response options including “not at all,” “several days,” “more than half the days,” and “nearly every day,” scored from 0 to 3. Higher scores indicate greater severity. A total score of ≥10 on the PHQ-9 is defined as depressive symptoms, with a sensitivity and specificity of 88% for screening moderate to severe depressive symptoms (20).

### Covariates

The selection of covariates is mainly based on demographics, lifestyle factors, and common chronic diseases. Demographics (21) include age, gender, race, marital status, education level, poverty income ratio (PIR), and BMI; lifestyle factors (22) include sleep status and smoking status; and common chronic diseases (23) include cardiovascular disease (CVD), hypertension, diabetes, and hyperlipidemia.

Race is categorized into four groups: Non-Hispanic White, Non-Hispanic Black, Mexican American or Other, while marital status is divided into three categories: Married/cohabiting, Never married, and Widowed/divorced/separated. Education level is classified into five categories: Less Than 9th Grade, 9–11th Grade, High School Grad/GED, Some College or AA Degree, College Graduate or above. Sleep status is based on the presence of sleep disorders. Smoking status is categorized into three groups: Never smoked, Former smoker, and Current smoker, with never smoked defined as smoking fewer than 100 cigarettes in a lifetime, former smoker defined as smoking more than 100 cigarettes but not currently smoking, and current smoker defined as smoking more than 100 cigarettes and currently smoking. PIR is categorized as ≤1.3, 1.3–3.5, and > 3.5. BMI is divided into <25, 25–30, and ≥ 30. CVD is defined based on the presence of congestive heart failure (CHF)/coronary heart disease (CHD)/angina/heart attack/stroke. Hypertension is determined by self-reported hypertension, or average systolic blood pressure ≥ 140 mmHg, or average diastolic blood pressure ≥ 90 mmHg, or use of antihypertensive medication. Diabetes diagnosis is based on physician diagnosis, use of antidiabetic medications, random/2-h oral glucose tolerance level ≥ 11.1 mmol/L, fasting blood glucose ≥7.0 mmol/L, or glycosylated hemoglobin (HbA1c) ≥6.5 mmol/L. Hyperlipidemia diagnosis includes HDL (male ≤40 mg/dL, female ≤50 mg/dL), triglycerides ≥150 mg/dL, total cholesterol ≥200 mg/dL, low-density lipoprotein ≥130 mg/dL, or use of cholesterol-lowering medications.

### Statistical analysis

Firstly, our study accounted for the complex sampling design and sample weights of the NHANES. Baseline characteristics of participants were described by gender, presented with numbers and percentages (%) for categorical variables and with mean ± standard error (SE) for continuous variables. Weighted *t*-tests were used for continuous variables, and weighted chi-square tests were used for categorical variables to compare significant differences in population characteristics.

We evaluated the association between ALI and the risk of depression using multivariable logistic regression models in the overall population as well as stratified by gender, with results presented as odds ratios (ORs) and 95% confidence intervals (CIs). We constructed regression models by stepwise adjustment of covariates: Model 1 adjusted for age, sex, and race; Model 2 further adjusted for smoking status, marital status, sleep status, education level, BMI, and PIR based on Model 1; Model 3 additionally adjusted for cardiovascular disease, hypertension, diabetes, and hyperlipidemia. Furthermore, we assessed the association between ALI, gender, and depression using mixed-effects binary logistic regression models, adjusting for the aforementioned covariates (24). Additionally, restricted cubic spline (RCS) models were separately fitted in the overall population and gender-stratified groups to explore the nonlinear relationship between ALI and depression.

Finally, to assess the stability of our findings, subgroup and interaction analyses were conducted separately in females and males using logistic regression models based on age (<60 or ≥ 60 years), smoking status (non-smoker or smoker), BMI (<25 or ≥ 25), diabetes (yes or no), hypertension (yes or no), dyslipidemia (yes or no), and CVD (yes or no). Moreover, there exists a significant association between gender and marital status, educational level, and depression (25). We investigated the interactions among gender, marital status, and educational level (26) to further explore the impact of gender differences on the development of depression. A statistical significance level was set at *p* < 0.05 (two-tailed). All statistical analyses were performed using R Studio (version 4.2.2).

## Results

### Baseline characteristics of the population

This cross-sectional study involved a weighted total of 20,849 adult participants, with the majority being non-Hispanic White individuals 10,017 participants (weighted proportion 71%). Among them, the numbers of female and male participants were roughly equal, with mean ages of 48 (17) years and 46 (16) years, respectively. Compared to male participants, females were mostly non-smokers (60 vs. 48%), had lower BMI (35 vs. 26%), a higher proportion of low-income individuals (22 vs. 19%), and a higher proportion with a college degree or above (33.7 vs. 30%). Additionally, the proportion of married or cohabiting individuals was lower among females compared to males (60 vs. 68%), while the proportion reporting no sleep disturbances was slightly higher (91.9 vs. 90.5%). Females also had a higher prevalence of hyperlipidemia, depression, and a lower prevalence of CVD (all *p* < 0.05) (Table 1).

**Table 1:** Characteristics of the study population, NHANES 2005–2018.

Characteristic	Overall, *N*^1^ = 20,849 (100%)^2^	Female, *N*^1^ = 10,335 (50.4%)^2^	Male, *N*^1^ = 10,514 (49.6%)^2^	*p*-value^3^
Age (years)	47 (17)	48 (17)	46 (16)	**<0.001**
Race				**<0.001**
*Non-Hispanic White*	10,017 (71%)	4,927 (71%)	5,090 (71%)	
*Non-Hispanic Black*	4,210 (10%)	2,110 (11%)	2,100 (9.4%)	
*Other Race*	3,462 (11%)	1,767 (11.1%)	1,695 (10.9%)	
*Mexican American*	3,160 (8.0%)	1,531 (6.9%)	1,629 (8.7%)	
PIR				**<0.001**
*High (>3.5)*	6,637 (43%)	3,121 (42%)	3,516 (46%)	
*Low (≤1.3)*	6,518 (21%)	3,404 (22%)	3,114 (19%)	
*Medium (1.3–3.5)*	7,694 (36%)	3,810 (36%)	3,884 (35%)	
BMI				**<0.001**
*Normal (<25)*	6,087 (31%)	3,199 (35%)	2,888 (26%)	
*Obese (≥30)*	7,766 (35%)	4,195 (37%)	3,571 (35%)	
*Overweight (25–30)*	6,996 (34%)	2,941 (28%)	4,055 (39%)	
Smoking status				**<0.001**
*Current smoker*	4,493 (21%)	1,909 (19%)	2,584 (24%)	
*Former smoker*	5,224 (25%)	2,023 (21%)	3,201 (28%)	
*Never smoker*	11,132 (54%)	6,403 (60%)	4,729 (48%)	
Education attainment				**<0.001**
*Less Than 9th Grade*	2,038 (5.0%)	919 (4.6%)	1,119 (5.6%)	
*9-11th Grade*	3,086 (11%)	1,511 (11%)	1,575 (11%)	
*High School Grad/GED*	4,802 (23%)	2,268 (21.8%)	2,534 (24%)	
*Some College or AA degree*	6,131 (32%)	3,288 (33.7%)	2,843 (30%)	
*College Graduate or above*	4,792 (29%)	2,349 (28.9%)	2,443 (29.4%)	
Marital status				**<0.001**
*Married/cohabiting*	12,501 (64%)	5,581 (60%)	6,920 (68%)	
*Never married*	3,655 (17%)	1,737 (16%)	1,918 (19%)	
*Widowed/divorced/separated*	4,693 (19%)	3,017 (24%)	1,676 (13%)	
Sleep disorders				**0.004**
*Yes*	1,794 (9.0%)	837 (8.1%)	957 (9.5%)	
*No*	19,055 (91%)	9,498 (91.9%)	9,557 (90.5%)	
CVD				**<0.001**
*Yes*	2,209 (8.0%)	932 (9.3%)	1,277 (8.3%)	
*No*	18,640 (92%)	9,403 (90.7%)	9,237 (91.7%)	
Hypertension				0.2
*Yes*	8,808 (37.5%)	4,344 (37%)	4,464 (38%)	
*No*	12,041 (62.5%)	5,991 (63%)	6,050 (62%)	
Diabetes				0.073
*Yes*	3,725 (13.3%)	1,785 (12.7%)	1,940 (13.8%)	
*No*	17,124 (86.7%)	8,550 (87.3%)	8,574 (86.2%)	
Hyperlipidemia				**0.037**
*Yes*	15,126 (71.9%)	7,670 (72.7%)	7,456 (71.1%)	
*No*	5,723 (28.1%)	2,665 (27.3%)	3,058 (28.9%)	
Depression				**<0.001**
*Yes*	19,007 (92.4%)	9,159 (90.3%)	9,848 (94.6%)	
*No*	1,842 (7.6%)	1,176 (9.7%)	666 (5.4%)	
Log2(ALI)	5.91 (0.69)	5.90 (0.69)	5.92 (0.70)	0.085

^1^*N* not Missing (unweighted); ^2^Mean ± SD for continuous(weighted); *n* (%) for categorical(weighted);^3^*t*-test adapted to complex survey samples; chi-squared test with Rao & Scott’s second-order correction. PIR, Poverty-income ratio; BMI, Body mass index; CVD, Cardiovascular disease; ALI, Advanced lung cancer inflammation index.

### Overall association between ALI levels and depression

The association between ALI levels and depression was evaluated through a multivariable logistic regression model. The results indicated a negative correlation between high and moderate ALI levels and the risk of depression. Specifically, after adjusting for age, gender, race, marital status, education level, smoking status, sleep quality, BMI, PIR, hypertension, diabetes, hyperlipidemia, and cardiovascular diseases, the prevalence of depression significantly decreased in populations with high and moderate ALI levels compared to low ALI levels (*p* for trend < 0.05). The prevalence decreased by 17% (OR, 0.83; 95% CI: 0.72–0.97) and 23% (OR, 0.77; 95% CI: 0.66–0.91), respectively (Table 2).

**Table 2:** Association between levels of ALI and depression.

	Log2(ALI)	Events	Model 1	Model 2	Model 3
Variable			OR (95% CI)	OR (95% CI)	OR (95% CI)
Overall	Tertiles				
	Low	6,816	Reference	Reference	Reference
	Medium	6,572	0.88(0.76, 1.01)	0.83(0.71, 0.97)^*^	0.83(0.72, 0.97)^*^
	High	7,461	0.88(0.74, 1.03)	0.76(0.65, 0.90)^**^	0.77(0.66, 0.91)^**^
	*p* for trend		*p* = 0.097	*p* = 0.002	*p* = 0.004

Model 1: adjusted for age, sex, and race. Model 2: adjusted for age, sex, race, PIR, BMI, sleep status, education level, marital status, and smoking status. Model 3: adjusted for age, sex, race, PIR, BMI, sleep status, education level, marital status, smoking status, cardiovascular disease, hypertension, diabetes, and hyperlipidemia. 95% CI, 95% Confidence interval; OR, Odds ratio; ALI; Advanced lung cancer inflammation index. ^*^p < 0.05, ^**^p < 0.01, ^***^p < 0.001; *p* < 0.05 was considered statistically significant.

### Association between ALI and depression among males and females

In our study on the association between ALI and depression, we found gender differences (Table 3). As we gradually adjusted for covariates, it appears that high ALI may benefit female individuals more compared to males. Specifically, among female participants, the results of the fully adjusted model show a significant negative correlation between the presence of depression and both the high ALI group (OR, 0.79; 95% CI: 0.64–0.98) and the mid ALI group (OR, 0.73; 95% CI: 0.60–0.88) compared to the reference low ALI group. However, we did not observe a significant association between ALI and depression in male participants, and there was an interaction between gender and ALI (*p* for interaction = 0.028). The results of the mixed-effects model indicate that, after adjusting for relevant covariates, the fixed effect variable of female gender is positively associated with a higher prevalence of depression compared to males (OR, 1.91; 95% CI: 1.73–2.09), while high Log2(ALI) levels, compared to low Log2(ALI), are negatively associated with the prevalence of depression (OR, 0.66; 95% CI: 0.46–0.88) (Supplementary Table S1). Moreover, high levels of Log2(ALI) can reduce the prevalence of depression in females (Supplementary Figure S2).

**Table 3:** Sex-specific association between ALI levels and depression.

Variable	Log2(ALI)	Events	Model 1	Model 2	Model3
			OR (95% CI)	OR (95% CI)	OR (95% CI)
Female	Tertiles				
	Low	3,329	Reference	Reference	Reference
	Medium	3,248	0.84 (0.70, 1.00)^*^	0.71 (0.59, 0.86)^***^	0.73 (0.60, 0.88)^**^
	High	3,758	1.00 (0.81, 1.23)	0.77 (0.62, 0.95)^*^	0.79 (0.64, 0.98)^*^
	*p* for trend		*p* = 0.997	*p* = 0.023	*p* = 0.048
Male	Tertiles				
	Low	3,487	Reference	Reference	Reference
	Medium	3,324	0.94 (0.72, 1.22)	1.06 (0.80, 1.40)	1.05 (0.79, 1.39)
	High	3,703	0.71 (0.55, 0.90)^**^	0.79 (0.62, 1.01)	0.78 (0.61, 1.01)
	*p* for trend		*p* = 0.004	*p* = 0.108	*p* = 0.108
*p* for interaction			*p* = 0.011	*p* = 0.027	*p* = 0.028

Model 1: adjusted for age, sex, and race. Model 2: adjusted for age, sex, race, PIR, BMI, sleep status, education level, marital status, and smoking status. Model 3: adjusted for age, sex, race, PIR, BMI, sleep status, education level, marital status, smoking status, cardiovascular disease, hypertension, diabetes, and hyperlipidemia. 95% CI, 95% Confidence interval; OR, Odds ratio; ALI; Advanced lung cancer inflammation index. ^*^p < 0.05, ^**^p < 0.01, ^***^p < 0.001; *p* < 0.05 was considered statistically significant.

**Figure 2:**
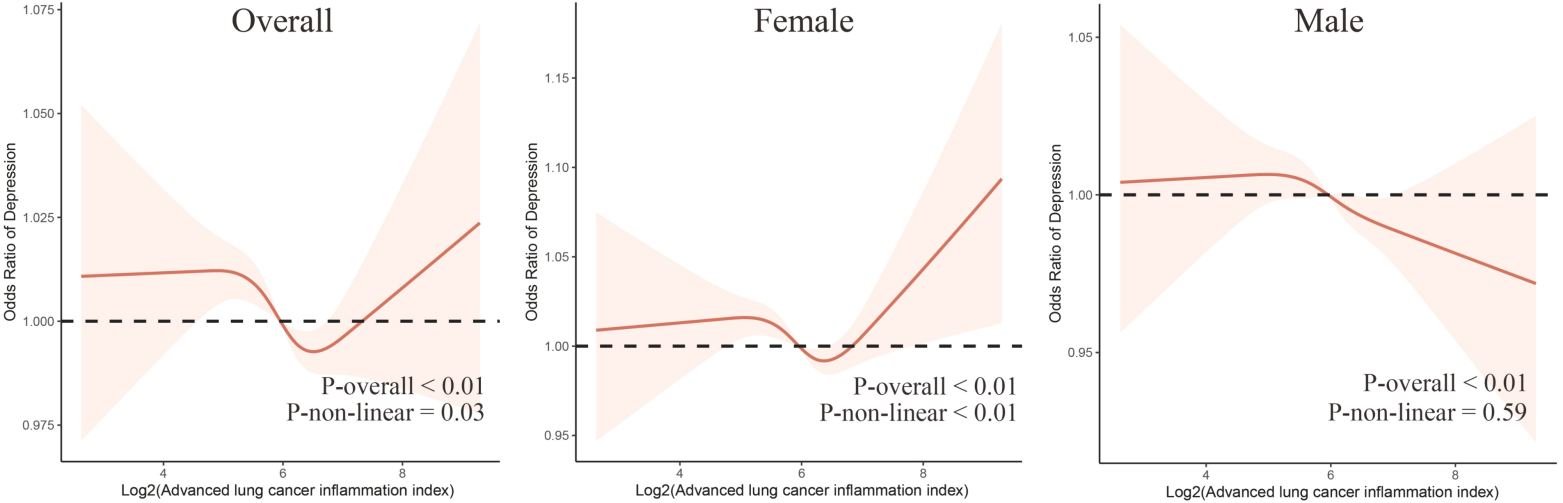
The dose–response relationship between Log2(ALI) and overall and sex-specific depression risk. The model has been adjusted for age, sex (excluding males and females themselves), race, marital status, education level, smoking status, sleep quality, BMI, PIR, hypertension, diabetes, hyperlipidemia, and cardiovascular diseases.

In addition, RCS analysis revealed curve differences between genders (Figure 2). A “U”-shaped non-linear relationship between log2(ALI) and depression was observed among all participants and female participants, while a linear relationship between log2(ALI) and depression was observed among males.

### Subgroup analyses

**Figure 3:**
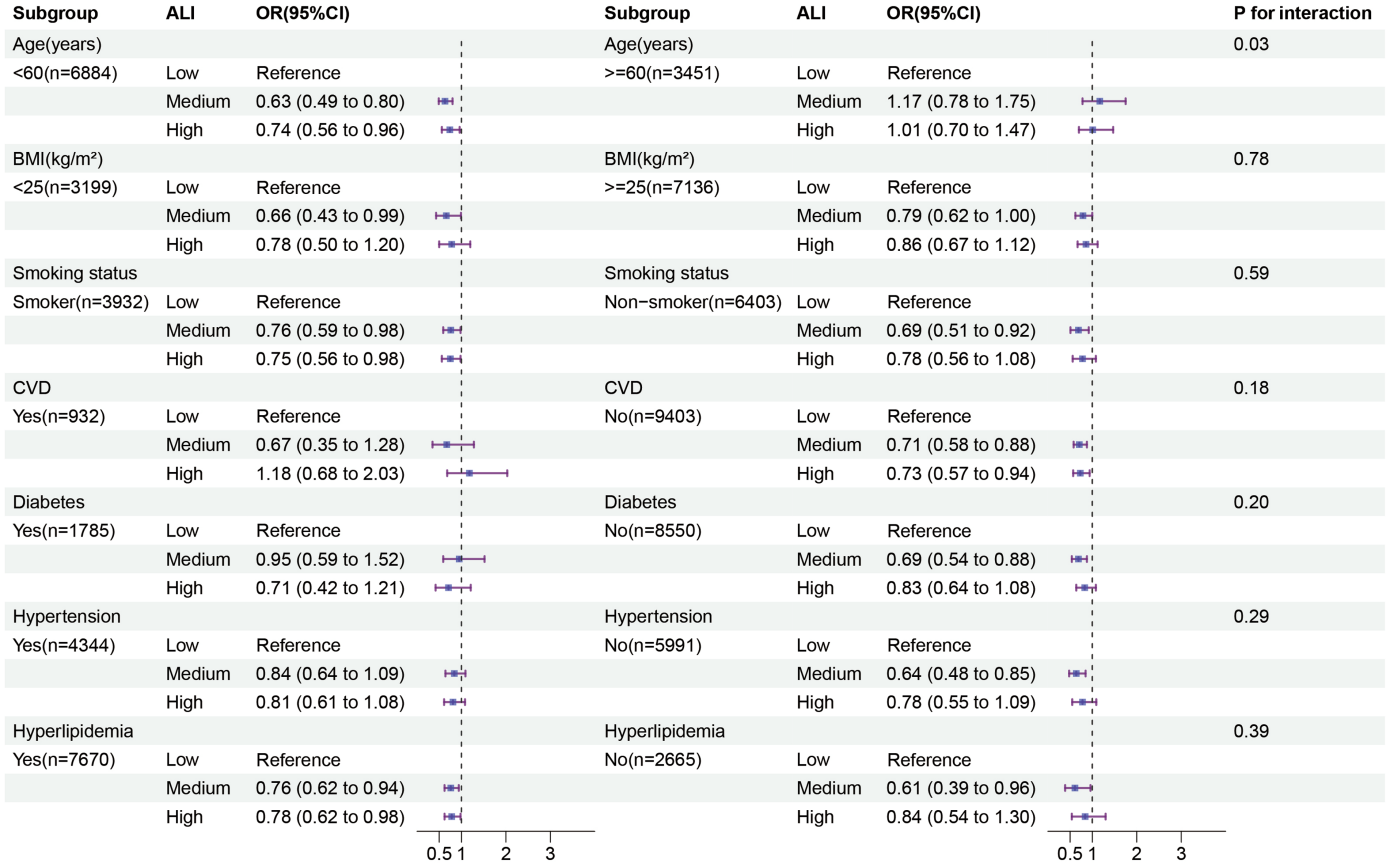
Subgroup analysis in the female group. The results of subgroup analysis were adjusted for all covariates except the effect modifier. BMI, Body mass index; CVD: Cardiovascular disease; ALI; Advanced lung cancer inflammation index.

We conducted stratified analyses separately in males and females. Among females, those aged <60 years without CVD, diabetes, or hypertension showed a more pronounced negative correlation between moderate to high levels of ALI and depression (Figure 3). Additionally, an interaction was found after stratification by age (*p* for interaction = 0.03), while no interactions were observed in the other stratified variables (all *p* for interaction >0.05). In males, those aged <60 years with diabetes or hypertension exhibited a more pronounced negative correlation between moderate to high levels of ALI and depression (Supplementary Figure S3). No interactions were observed in the stratified analyses (all *p* for interaction >0.05). In the additional analysis of the interaction between gender, marital status, and education level (Supplementary Table S2), we found that females are more susceptible to depression compared to males. Moreover, higher levels of education are associated with a lower likelihood of depression. Furthermore, individuals who are single, widowed, or separated exhibit a higher risk of depression compared to their married counterparts. Importantly, we observed a significant interaction between gender and marital status (*p* for interaction = 0.03).

## Discussion

In this study, we analyzed the association between ALI and depression prevalence in the general population as well as between genders. We found a negative and non-linear relationship between ALI and depression prevalence in the overall United States population. Further gender-specific analysis revealed a more pronounced association among females, which was confirmed by mixed-effects logistic regression models. Additionally, subgroup analysis indicated a more significant negative correlation between moderate to high ALI levels and depression risk among females under the age of 60.

There is an interplay between inflammation and nutrition, with some nutrients and other food components having anti-inflammatory potential, such as Omega-3 and Omega-6 fatty acids (27, 28). Pro-inflammatory cytokines like interleukin 6 (IL-6) and interleukin 1β (IL-1β) interact with glucagon-like peptide-1 (GLP-1) released by intestinal tissues, leading to decreased food intake and unintended weight loss (29). Our study comprehensively investigates, for the first time, the combined effects of inflammation and nutrition on depression, and we found gender differences in the impact of inflammation and nutrition on depression. Unlike previous single inflammatory or nutritional markers, the ALI score consists of three factors: BMI, serum albumin levels, and the NLR, where BMI and albumin levels reflect nutritional status (30, 31), and NLR reflects inflammatory status (32), making ALI a comprehensive assessment index based on both nutrition and inflammation. Firstly, depression has been associated with inflammation in various reports (33, 34), where neutrophils serve as frontline innate immune defenders, crucially recruiting immune cells to inflammatory sites (35), while lymphocytes mainly mediate adaptive immunity, exerting regulatory or protective functions, and low lymphocyte counts generally indicate poor condition (36, 37). NLR thus reflects two different immune pathways and can better reflect inflammatory status. Demircan et al. (38) included 80 Major depressive disorder patients, showing significantly higher NLR in depression patients compared to controls, and a meta-analysis indicated a significant association between higher NLR and increased risk of depression (39). Previous reports have indicated that females have higher baseline abundance of neutrophils and are more sensitive to the pro-inflammatory signals they produce (40). Additionally, there is a communication mechanism involving the metabolic hormone leptin between neutrophils and the brain (41). Due to higher levels of adipose tissue in females, the generation rate of leptin is faster (42). The increase in leptin concentration makes it more likely for neutrophils in females to migrate from the periphery to the central nervous system to activate the immune circuit (43).

Serum albumin is a multifunctional plasma protein with important antioxidant properties. According to previous reports (11), the release of pro-inflammatory cytokines leads to dysregulation of the HPA axis mechanism, resulting in enhanced oxidative stress, further causing oxidative damage associated with neurodegeneration and various mental disorders (including depression) (44). A retrospective study from Taiwan showed that serum albumin levels in the normal control group were significantly higher than those in patients with severe depression (45), and additionally, studies have found abnormally high levels of oxidative products in the peripheral blood, red blood cells, and cerebrospinal fluid of individuals with severe depression (46, 47). Daniel McFarland’s research further suggests that albumin assessment can help identify the presence of inflammation in the management of depression, anxiety, and related psychological symptoms in patients with metastatic cancer (48). BMI is a fundamental measure of body fat content. Numerous studies have shown that to some extent, an increase in BMI is associated with increased severity of depression (49, 50). This may be attributed to the pursuit of an idealized lower weight, leading individuals who are overweight or obese to experience self-esteem issues, including dissatisfaction with their bodies, which is more prominent in females and is believed to increase the risk of depression (51). Furthermore, the direct and indirect impacts of emotional eating on BMI are more pronounced in females (52). Additionally, according to our findings, higher levels of albumin and BMI, as well as lower NLR, imply a lower risk of depression. Moreover, RCS results suggest a “U”-shaped relationship between ALI and depression prevalence, which may be BMI-driven. Consistent with previous studies, excessive increase in BMI within ALI is associated with increased risk of depression, possibly involving leptin, which can activate neurogenesis in the hippocampus, promoting its antidepressant-like behavioral effects (53). In obese patients, however, levels of leptin are decreased, leading to impaired glucocorticoid-mediated negative feedback of the HPA axis, resulting in lack of pleasure and social aversion (54). Furthermore, a previous study in a Chinese population also demonstrated a significant “U”-shaped trend between BMI and depression (55), consistent with the direction of our RCS results.

For depression, most studies seem to overlook gender differences. Our research found that the risk of depression is greater in females than in males, and the relationship between ALI and reducing the risk of depression is more pronounced in females, while it becomes nonsignificant in males. This gender difference can be explained by sex hormones between males and females. Firstly, females undergo significant hormonal changes multiple times throughout life, especially during the menstrual cycle, pregnancy, postpartum, and menopause (56–58). Fluctuations in estrogen and progesterone levels during these periods, particularly sharp changes in estrogen levels, are believed to be associated with increased risk of depression (59, 60). For example, during certain stages of the menstrual cycle, such as premenstrual and menstrual phases, mood issues and depressive symptoms may worsen due to a decrease in estrogen levels (56). Additionally, female brain structure and function are more sensitive to hormonal fluctuations (61). Fluctuations in estrogen levels can alter hippocampal neuroplasticity, affecting mood and cognitive function, thereby increasing the risk of depression (62). Of course, some genetic factors, such as brain-derived neurotrophic factor (BDNF) gene, may interact with sex hormones, making females carrying specific genetic variants more susceptible to depression during hormonal fluctuations (63). Compared to females, males have higher levels of testosterone, which has been shown to increase phagocytic activity while decreasing the antimicrobial activity of neutrophils (64). It also possesses antioxidant properties. Additionally, chronic stress leads to excessive activation of the HPA axis, resulting in excess glucocorticoids and impaired negative feedback, contributing to stress-related mental illnesses including depression (65), whereas testosterone can inhibit the activity of the HPA axis (66). Furthermore, our study findings indicate an interaction between gender and marital status. Specifically, individuals who are single, widowed, or separated exhibit a higher risk of depression compared to their married counterparts, consistent with previous research (67). The care and support of a spouse are crucial pillars in mitigating feelings of loneliness and reducing the risk of depression (68). However, overall, females are still more prone to depression than males. In cases where females face conflicts between their family and work roles post-marriage, they often prioritize their domestic roles over their professional ones, leading to missed opportunities for career advancement, thereby reducing their social status and self-worth, significantly increasing their risk of depression (69).

The research findings can offer suggestions for policymakers and researchers. At the policy level, integrating ALI into routine screening for depression is advisable, as it is easy to calculate and cost-effective. Strengthening health education through community, healthcare, and educational institutions to emphasize the relevant information on nutrition and inflammation is meaningful, particularly for the female population, to prevent potential symptoms of depression. In the future, more long-term studies are needed to validate the causal relationship between ALI and depression. This study has several strengths. Firstly, it is based on a large-scale sample that is nationally representative. Secondly, it comprehensively evaluates the association between nutritional and inflammatory status and the risk of depression for the first time, while also exploring gender differences in this relationship. However, we acknowledge some limitations. Firstly, our study is cross-sectional, thus causal inferences cannot be made. Secondly, depression was assessed through self-report using the PHQ-9, which may introduce recall bias, and depression symptoms range from mild to severe, which may have different relationships with ALI. Thirdly, despite controlling for key demographic indicators, behavioral risk factors, and various chronic diseases, the results may still be influenced by unknown confounding factors. Fourthly, this study was conducted on a representative sample in the United States, so the generalizability of the research findings may be limited. It is necessary to recruit participants from different cultural or geographical backgrounds for multicenter clinical trials.

## Conclusion

In this study, conducted among a nationally representative sample of adult Americans, we observed a negative correlation between moderate to high levels of ALI and the prevalence of depression, along with gender differences. Specifically, in females, greater attention should be given to nutritional and inflammatory status. Furthermore, these findings provide new insights for researchers and clinical practitioners to develop more effective intervention strategies and policies.

## Data availability statement

The original contributions presented in the study are included in the article/Supplementary material, further inquiries can be directed to the corresponding author.

## Author contributions

YX: Formal analysis, Investigation, Software, Validation, Writing – original draft. ZY: Investigation, Software, Validation, Writing – original draft. LL: Funding acquisition, Supervision, Visualization, Writing – review & editing.
