# Supplementary material for: Sex differences in the combined influence of inflammation and nutrition status on depressive symptoms: insights from NHANES
Source: Front Nutr. 2024 May 29;11:1406656. doi: 10.3389/fnut.2024.1406656 (PMC11168495; doi:10.3389/fnut.2024.1406656)
Supplement: Supplementary file 1 [file Data_Sheet_1.docx]

Table S1 The results of the mixed-effects binary logistic regression model.

|  | **Mixed effects binary logistic regressions** | **Depression** | |
| --- | --- | --- | --- |
| **Crude Model** | **Fixed effects** | **OR (95% CI)** | **P-value** |
|  | **Gender** | |  |
|  | Men | Reference |  |
|  | Women | 1.71(1.54,1.88) | **<0.001** |
|  | **Log2(ALI)-Tertiles** | |  |
|  | Low | Reference |  |
|  | Medium | 0.95(0.76,1.34) | >0.05 |
|  | High | 0.73(0.54,0.92) | **<0.01** |
| **Adjust Model** | **Fixed effects** | **OR (95% CI)** | **P-value** |
|  | **Gender** |  |  |
|  | Men | Reference |  |
|  | Women | 1.91(1.73,2.09) | **<0.001** |
|  | **Log2(ALI)-Tertiles** |  |  |
|  | Low | Reference |  |
|  | Medium | 0.93(0.74,1.13) | >0.05 |
|  | High | 0.66(0.46,0.88) | **<0.01** |

95% CI, 95% confidence interval; OR, odds ratio; ALI, Advanced Lung Cancer Inflammation Index.

Crude Model: Unadjusted model; Adjust Model: adjusted for age, race, PIR, BMI, sleep status, education level, Marital status, smoking status, cardiovascular disease, hypertension, diabetes and hyperlipidemia.

Table S2 Interactions of Gender, Marital Status, and Education Level with Depression.

|  | **OR (95%CI)** | **P-value** |
| --- | --- | --- |
| **Marital status, Female** |  |  |
| Married/cohabiting | Reference |  |
| Never married | 0.98 (0.75~1.29) | 0.90 |
| Widowed/divorced/separated | 1.62 (1.30~2.02) | **<0.001** |
| **Marital status, Male** |  |  |
| Married/cohabiting | Reference |  |
| Never married | 1.67 (1.23~2.26) | **0.001** |
| Widowed/divorced/separated | 2.16 (1.70~2.74) | **<0.001** |
| **Sex, Married/cohabiting** |  |  |
| male | Reference |  |
| female | 2.54(2.10~3.08) | **<0.001** |
| **Sex, Never married** |  |  |
| male | Reference |  |
| female | 1.50(1.09~2.07) | **0.014** |
| **Sex, Widowed/divorced/separated** |  |  |
| male | Reference |  |
| female | 1.52(1.14~2.02) | **0.005** |
| **Sex: Marital status** | - | **0.03** |
| **Education attainment, Female** |  |  |
| Less Than 9th Grade | Reference |  |
| 9-11th Grade | 0.84 (0.62~1.13) | 0.20 |
| High School Grad/GED | 0.60 (0.44~0.82) | **0.002** |
| Some College or AA degree | 0.62 (0.44~0.86) | **0.005** |
| College Graduate or above | 0.39 (0.26~0.61) | **<0.001** |
| **Education attainment, Male** |  |  |
| Less Than 9th Grade | Reference |  |
| 9-11th Grade | 0.75 (0.52~1.08) | 0.12 |
| High School Grad/GED | 0.84 (0.56~1.26) | 0.40 |
| Some College or AA degree | 0.75 (0.51~1.10) | 0.13 |
| College Graduate or above | 0.41 (0.24~0.68) | **<0.001** |
| **Sex, Less Than 9th Grade** |  |  |
| male | Reference |  |
| female | 1.87(1.17~3.00) | **0.010** |
| **Sex, 9-11th Grade** |  |  |
| male | Reference |  |
| female | 2.56(1.96~3.36) | **<0.001** |
| **Sex, High School Grad/GED** |  |  |
| male | Reference |  |
| female | 1.67(1.96~3.36) | **0.001** |
| **Sex, Some College or AA degree** |  |  |
| male | Reference |  |
| female | 1.67(1.23~2.27) | **<0.001** |
| **Sex, College Graduate or above** |  |  |
| male | Reference |  |
| female | 1.87(1.43~2.44) | **<0.001** |
| **Sex: Education attainment** | - | 0.26 |

95% CI, 95% confidence interval; OR, odds ratio; The p-values for the interactions between Sex and Marital Status, with Sex and Education Attainment were calculated using ANOVA (analysis of variance). Adjusted for age, race, PIR (Poverty Income Ratio), BMI (Body Mass Index), sleep status, marital status, education level, smoking status, cardiovascular disease, hypertension, diabetes, and hyperlipidemia, with mutual adjustments for marital status and education level.


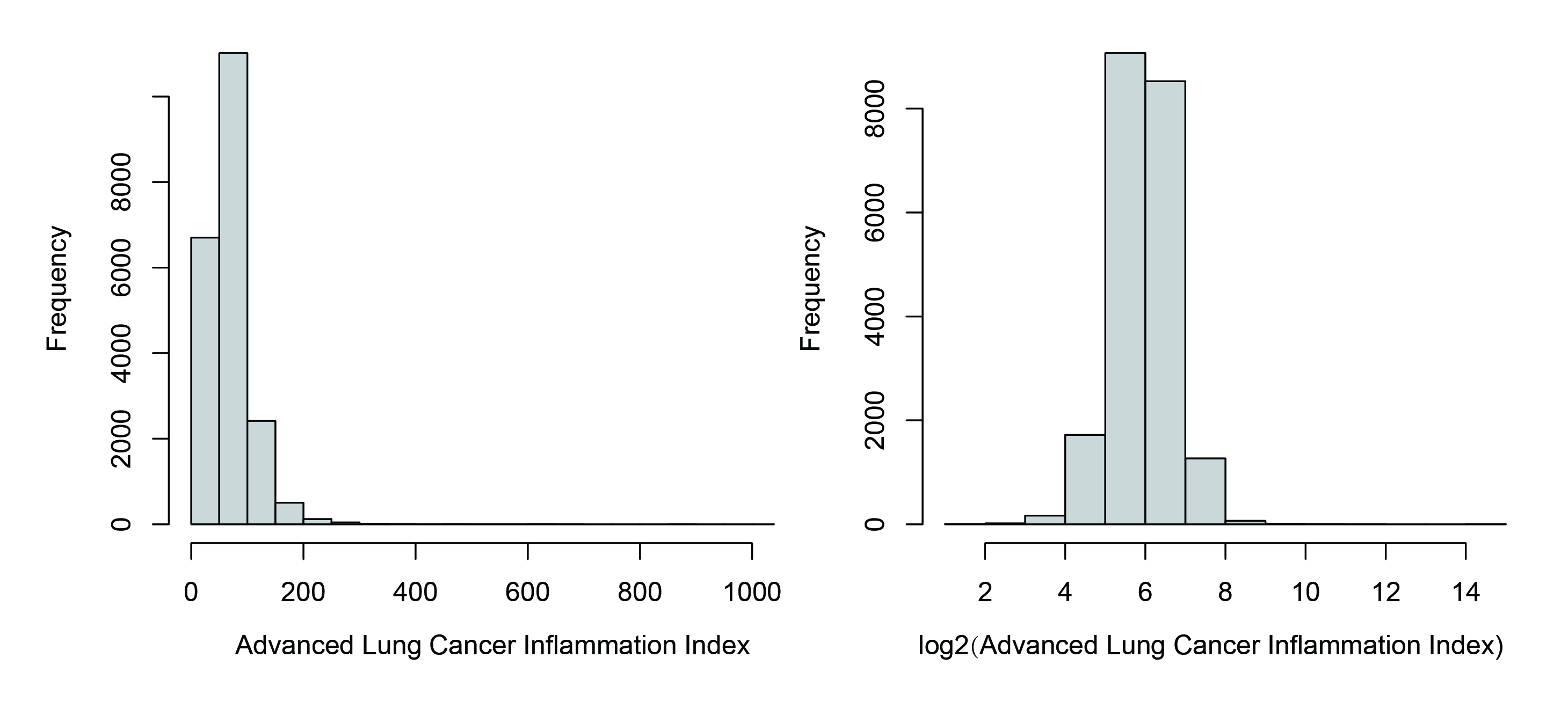


Figure S1 The distribution of the Advanced Lung Cancer Inflammation Index and the distribution of the log2-transformed Advanced Lung Cancer Inflammation Index.


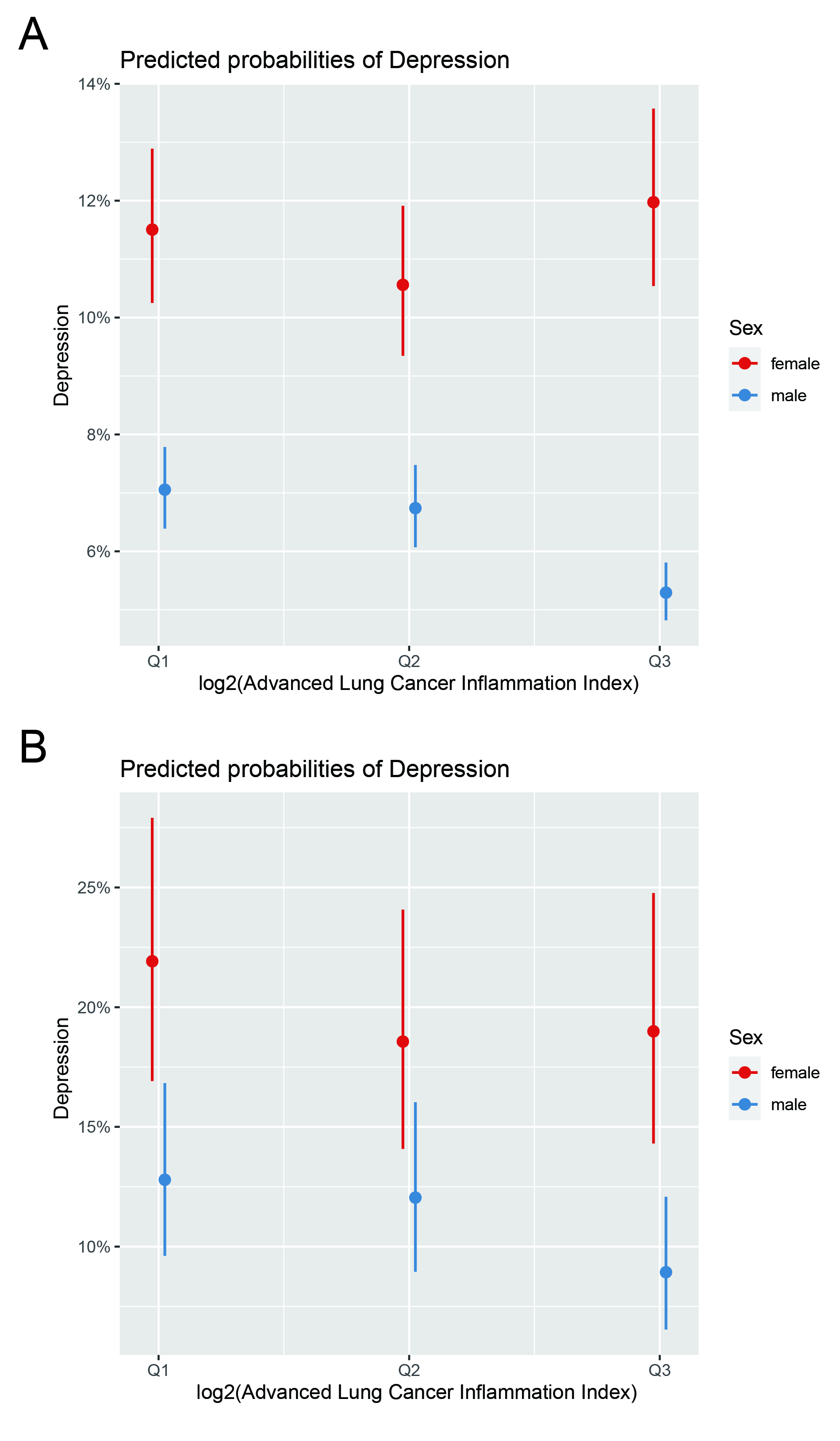


Figure S2 Mixed effects binary logistic regressions. A. Unadjusted model; B. adjusted for age, race, PIR, BMI, sleep status, education level, Marital status, smoking status, cardiovascular disease, hypertension, diabetes and hyperlipidemia.


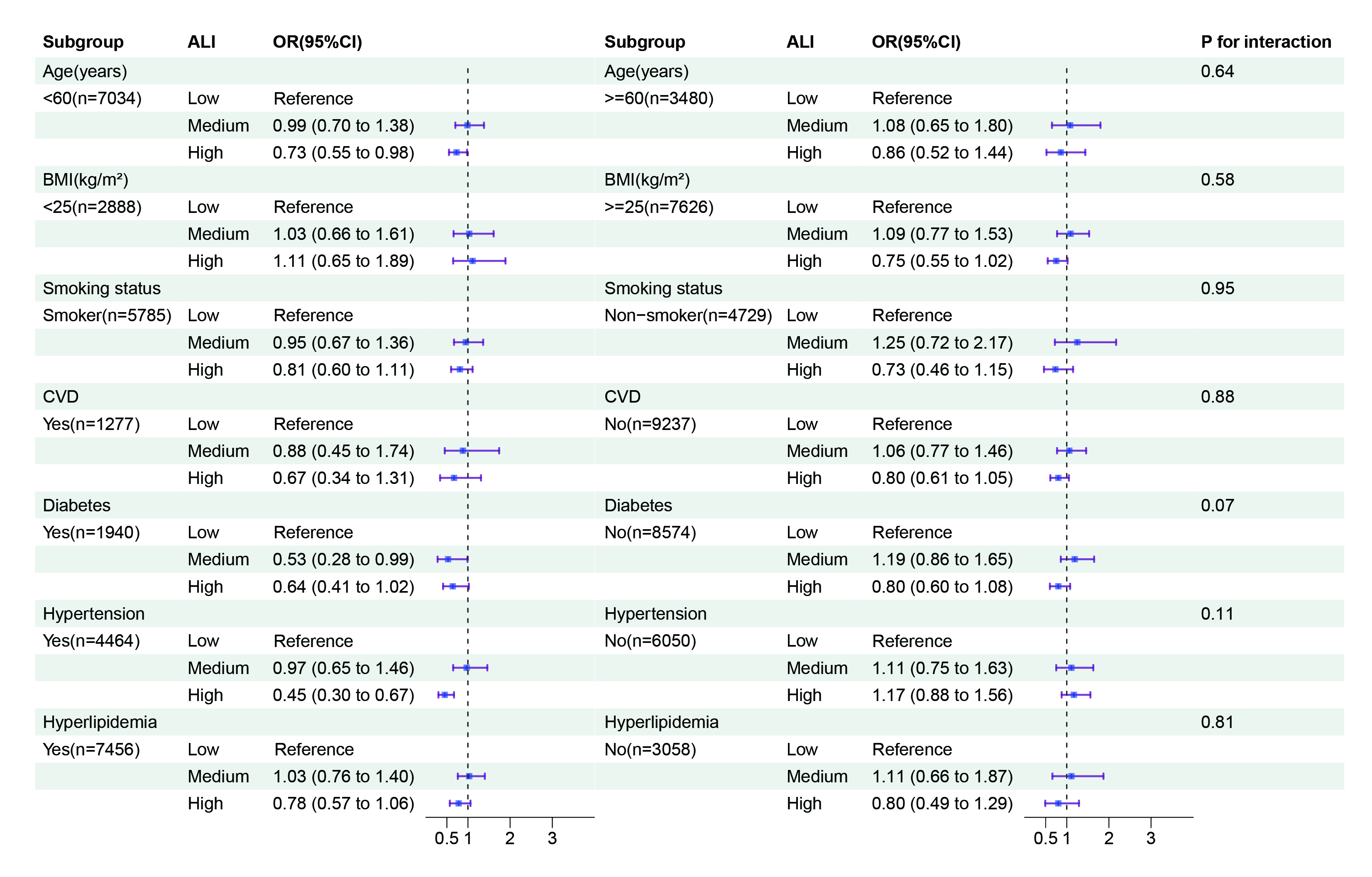


Figure S3 Subgroup analysis in the male group.

BMI, Body Mass Index; CVD: Cardiovascular Disease; ALI; Advanced Lung Cancer Inflammation Index.
